# Prognostic Factors for Immune Thrombocytopenia Outcome in Greek Children: A Retrospective Single-Centered Analysis

**DOI:** 10.1155/2017/7878605

**Published:** 2017-12-06

**Authors:** Alexandros Makis, Athanasios Gkoutsias, Theodoros Palianopoulos, Eleni Pappa, Evangelia Papapetrou, Christina Tsaousi, Eleftheria Hatzimichael, Nikolaos Chaliasos

**Affiliations:** ^1^Department of Pediatrics, University Hospital of Ioannina, Ioannina, Greece; ^2^Department of Internal Medicine, University Hospital of Ioannina, Ioannina, Greece; ^3^Hematology Laboratory, University Hospital of Ioannina, Ioannina, Greece; ^4^Hematology Department, University Hospital of Ioannina, Ioannina, Greece

## Abstract

Immune thrombocytopenia (ITP) in children has a varied course and according to duration is distinguished as newly diagnosed (<3 months), persistent (3–12), and chronic (>12) types. Several studies have evaluated the prognostic factors for the progression of the disease, but similar works have yet to be performed in Greece. We aimed to identify prognostic markers for the three forms of the disease in 57 Greek children during a 13-year period. Information regarding age, gender, preceding infection, bleeding type, duration of symptoms and platelets at diagnosis, treatment, disease course, and immunological markers was recorded. 39 children had newly diagnosed, 4 persistent, and 14 chronic disease. Chronic ITP children were more likely to be of age > 10 years (*p* = 0.015) and have gradual initiation of the disease (*p* = 0.001), platelets > 10 × 10^9^/L (*p* = 0.01), and impaired immunological markers (*p* < 0.003) compared to newly diagnosed/persistent groups. Recent history of infection was found mainly in the newly diagnosed/persistent group (*p* = 0.013). None of the children exhibited severe spontaneous bleeding.* Conclusion*. Even though ITP in children usually has a self-limited course, with rare serious bleeding complications, the chronic form of the disease is characterized by different predictive parameters, which can be used in clinical practice.

## 1. Introduction

Primary immune thrombocytopenia (ITP) is an autoimmune disorder characterized by low platelet count (<100 × 10^9^/L) in the absence of other secondary causes. The disease is caused by increased platelet destruction by humoral or cellular immune mechanisms as well as inappropriate platelet production in the bone marrow [1]. In children, it mainly occurs between 2 and 7 years of age and usually a viral infection of the respiratory or gastrointestinal tract precedes 2–4 weeks earlier. At older ages, the precipitating factor of immune deregulation remains unknown [2].

ITP is characterized by a variety of skin and mucous membrane bleeding manifestations such as petechiae, purpura, bruising, epistaxis, gingival bleeding, and menorrhagia. Severe intracranial bleeding is extremely rare, occurring in 0.5–1% of children when the platelet count drops below 10 × 10^9^/L [3]. According to the standardization of terminology, definitions and outcome criteria in ITP of adults and children published by Rodeghiero et al., ITP in children is divided into newly diagnosed (duration < 3 months, 50% of the cases), persistent (3–12 months, 25% of the cases) and chronic (>12 months, 25% of the cases) type. The older time limit of 6 months to define chronicity is no longer in use [4].

Children who have no or mild bleeding can be managed with observation alone, regardless of platelet count. In cases requiring treatment, intravenous immunoglobulin (IVIG), anti-D immunoglobulin, or corticosteroids can be administered [5]. When, despite repeated doses of first-line treatment, the disease becomes resistant and chronic lasting more than twelve months, other treatments should be applied. Recently, the use of the thrombopoietin receptor agonist eltrombopag has been approved in children older than one year with chronic ITP who have not responded to the administration of first-line drugs [6]. In refractory cases, other options are the use of rituximab, a monoclonal anti-CD20 chimeric antibody, or immunosuppressant drugs (e.g., cyclosporine, azathioprine, and mycophenolate mofetil) [7]. Splenectomy is an alternative choice with a significant risk of complications and is applied only in a few, very serious chronic cases [8].

Reliable prediction of the course of the disease at time of diagnosis could be a useful tool regarding the planning of treatment, in order to minimize the risk of bleeding while avoiding drug complications. Also, it helps the patients and their parents to know what to except in the future and to cope with the changes that inevitably arise in the life of the child and the family.

The predictors of the progression of the disease have been investigated in large, international studies with focus on the distinction between acute and chronic form using the time limit of 6 months from diagnosis [9–11]. In recent studies, an effort has been made to determine the predictive parameters based on the new classification in which the time limit for chronic ITP is 12 months [12–14]. A recent meta-analysis included all studies and showed that chronic ITP is mainly correlated with the female gender, older age at diagnosis, absence of recent infection, slow onset of symptoms, higher platelet count at diagnosis, the presence of antinuclear antibodies (ANA), and treatment with corticosteroids and IVIG [15].

Since ITP is a heterogeneous disease, it is of great importance to confirm these ITP predictors at national level. In Greece, one retrospective work has been published with a large number of children from Crete and it was mainly focused on the treatment and the response to it [16]. The purpose of our study was to analyze all the ITP cases from an academic reference center in Northwestern Greece during a 13-year period and to point out the specific predictive characteristics related to the three categories of ITP (newly diagnosed, persistent, and chronic).

## 2. Patient and Methods

The medical records of all children with ITP hospitalized in the Pediatric Department of the University Hospital of Ioannina in Greece, from November 2002 to March 2015, were retrospectively studied. The Pediatric Department is the referral center for pediatric diseases in Northwestern Greece, with a mean number of 3.450 admissions per year. The demographic, clinical, and laboratory data of patients were recorded. The study was approved by the local ethics committee. The diagnosis of primary ITP was set in children with isolated thrombocytopenia (<100 × 10^9^/L) in the absence of other causes that may be associated with thrombocytopenia. Children with secondary thrombocytopenia due to systemic disease or medications, patients with incomplete clinical data, or patients that discontinued monitoring in our department were excluded from the study. All children had a minimum of 1 year of follow-up.

Demographic data were collected such as name, date of birth, age, sex, and place of residence. Medical and family history were recorded. The following information was documented at diagnosis: date of diagnosis, platelet count, type and severity of bleeding, and immunological markers (i.e., ANA, antiphospholipid antibodies, immunoglobulin levels, C3 and C4 levels, and direct antiglobulin test). We also recorded the type of treatment and the response.

Newly diagnosed ITP was defined as the presence of thrombocytopenia for <3 months after diagnosis, persistent for 3–12 months, and chronic ITP for >12 months. The onset of symptoms was defined as abrupt when bleeding symptoms lasted for less than two weeks before seeking assistance or as gradual when they lasted more than two weeks before the medical assessment. Platelet number at diagnosis was classified into <10 × 10^9^/L and >10 × 10^9^/L. This value was chosen because below this threshold spontaneous and serious bleeding manifestations can occur. A history of recent infection was defined as a recorded infection of the upper respiratory or gastrointestinal tract over four weeks before the diagnosis of ITP.

The statistical analysis was done using the SPSS 16.0 statistical program. Numerical data and categorical variables were analyzed by the Mann–Whitney* U* or *t*-tests and the chi-square test, respectively. The odds ratio (OR) and 95% confidence interval (CI) were used to determine the increased relative risk. *p* values less than 0.05 were considered statistically significant.

## 3. Results

**Figure 1:**
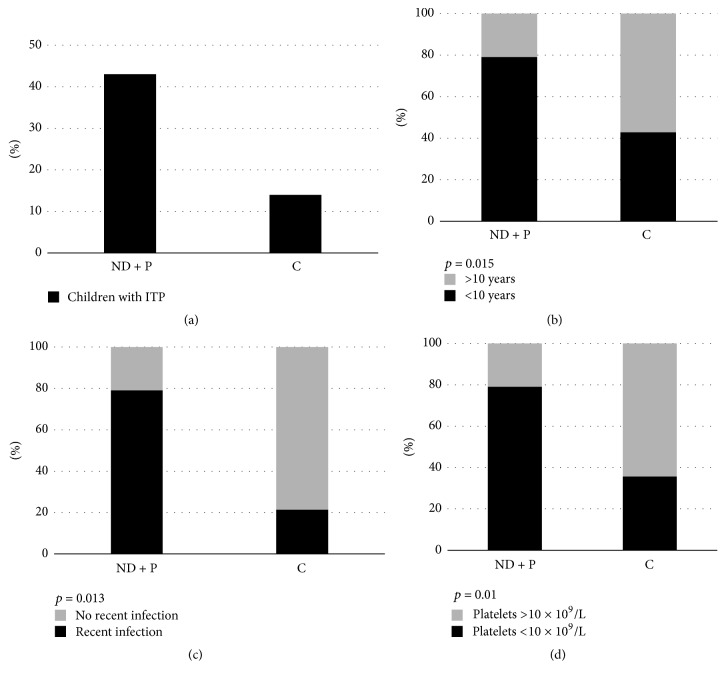
(a) Number of children with newly diagnosed/persistent (ND + P) and chronic (C) ITP, (b) percentage of children < 10 years or >10 years of age, (c) percentage of children with a history of recent infection or not, and (d) percentage of children with platelets less or more than 10 × 10^9^/L, at diagnosis.

A total of 57 children diagnosed with ITP were recorded during the study period. 39 (68%) children had newly diagnosed, 4 (7%) persistent, and 14 (25%) chronic form (Figure 1(a)). Due to the small number of children with persistent form, we decided to incorporate them to the group of children with newly diagnosed ITP. The characteristics of the newly diagnosed/persistent group were compared with the chronic ITP group. The findings are summarized in Table 1.

The age range of the patients varied from 1 from 16 years with a median value of 5.2 years. 42 of 57 children (74%) were aged <10 years. Regarding the children with newly diagnosed/persistent disease, the median age was 4.8 (range 1–12) and 34 of 43 (79%) were aged <10 years. In the chronic disease group, the median age was 11.3 (range 8–16) and 6 of 14 (43%) were below 10 years. The comparison between the two groups revealed a statistically significant result (*p* = 0.015), meaning that children <10 years of age were more likely to have newly diagnosed/persistent form (Figure 1(b)).

Of the 57 children, 27 were girls (47%) and 30 boys (53%). In the newly diagnosed/persistent disease group, 23 (53%) children were boys, while 7 (50%) children were boys in the chronic disease group, a nonstatistically significant difference (*p* = 0.72).

Recent infection history was recorded in 34 (79%) children with newly diagnosed/persistent disease and in 3 (21%) from the chronic ITP group, which was a statistically significant result (*p* = 0.013). This means that children with newly diagnosed/persistent disease had more often a history of preceding infection (Figure 1(c)).

The median platelet count at diagnosis was 14.6 × 10^9^/L (range 0–52) in newly diagnosed/persistent type and 26.3 × 10^9^/L (range 0–92) in chronic ITP. Platelets below 10 × 10^9^/L were observed in 34 of 43 children with newly diagnosed/persistent type (79%), while the same percentage was lower in children with chronic ITP (5/14, 36%) (*p* = 0.01) (Figure 1(d)).

**Figure 2:**
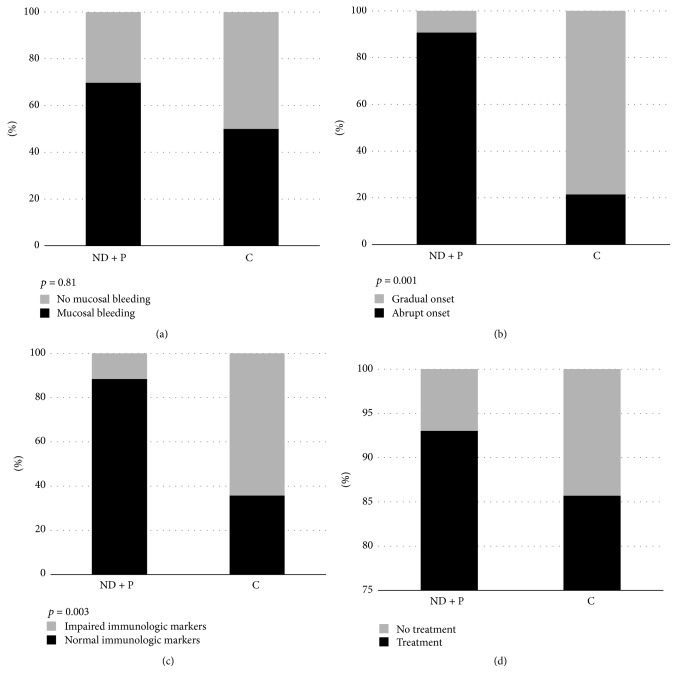
(a) Percentage of children with newly diagnosed/persistent (ND + P) and chronic (C) ITP with mucosal bleeding at diagnosis or not, (b) percentage of children with abrupt (<2 weeks) or gradual (>2 weeks) symptomatology before diagnosis, (c) percentage of children with normal or impaired immunological markers, and (d) percentage of children who received or did not receive treatment with intravenous immunoglobulin and/or corticosteroids.

Mucosal bleeding was observed in 37 of 57 patients (65%). In the newly diagnosed/persistent group, 30 of 43 children (70%) had mucosal bleeding, while the same number in chronic ITP group was 7/14 (50%), a nonsignificant result (*p* = 0.81) (Figure 2(a)). Only one 5-year-old boy (1 of 57, 1.75%) with newly diagnosed ITP had severe bleeding, namely, subdural hematoma. However, it was not a spontaneous bleeding, but occurred after an accidental fall and head injury. The outcome was excellent.

Concerning the onset of symptoms, the disease occurred abruptly in 39 of 43 (91%) of the cases with newly diagnosed/persistent form. In contrast, 3 of 14 (21%) children with chronic disease had abrupt onset, a statistically significant difference (*p* = 0.001) (Figure 2(b)).

Regarding immunological markers, 5 of 43 children (12%) with newly diagnosed/persistent disease had pathological results. On the contrary, chronic ITP children had more often (9 of 14 children, 65%) impaired immunological markers (*p* < 0.003) (Figure 2(c)). One 11-year-old boy with chronic disease was finally diagnosed as Evans syndrome, with positive direct antiglobulin test and markers of hemolysis, two years after diagnosis.

In total, 52 of 57 children (91%) received treatment with IVIG and/or corticosteroids sometime during the course of the disease. The percentage of children with newly diagnosed/persistent disease that required treatment was greater than those with chronic disease [40/43 (93%) versus 12/14 (86%)], but the difference was not statistically significant (*p* = 0.78) (Figure 2(d)).

Among the potential predicting factors for developing chronic ITP at diagnosis, the most significant predictors were found to be gradual onset (OR = 3.8, CI = 1.6–5.7), negative history of recent infection (OR = 3.1, CI = 1.8–4.9), age of more than 10 years (OR = 2.8, CI = 1.5–4.6), platelet count > 10 × 10^9^/L (OR = 2.1, CI = 1.2–4.1), and abnormal immunological markers (OR = 1.7, CI = 1.1–3.2).

## 4. Discussion

The present study investigated the parameters at diagnosis that distinguish the newly diagnosed/persistent from chronic ITP. According to the results, chronic ITP children are more likely to be of age older than 10 years and have negative history of recent infection, longer duration of symptoms, platelet count above 10 × 10^9^/L, and impaired immunological markers.

The age of the patients at diagnosis has been investigated as a predictor factor in several studies with large number of patients. The investigators of the Intercontinental Childhood ITP Study Group observed that chronic ITP was more frequently found in children above 10 years of age (47.3%) rather than infants (23.1%) [9]. In another large, multicenter study, it was noticed that children at the age of 10 years were at higher risk for developing chronic ITP than at the age of 2 years [10]. In our study, we observed that children with newly diagnosed/persistent ITP were mainly under 10 years and in children with chronic disease the same proportion was significantly lower. Therefore, increased age, especially adolescence, seems to be associated with chronicity. Also, in our study, all forms of the disease showed a similar incidence in both genders, a finding that is in accordance with previous studies [17]. Of notice, the age range of our patients did not include infants.

Preceding infection has been reported as a frequent finding in childhood ITP. In a large single center study, history of recent infection at diagnosis was reported in 56% of newly diagnosed/persistent disease cases. In contrast, 77% of patients with chronic disease had no history of preceding infection [18]. In a population-based, cohort study of the predictors of chronic ITP, it was found that patients with a history of recent infection at diagnosis were less likely to develop chronic ITP than patients without a relevant history (relative risk 0.44) [10]. In our patient analysis, the findings were similar. The most common viral agents that cause infectious diseases in childhood can potentially cause a transient immunological deregulation that leads to the production of antiplatelet antibodies and ITP with acute and self-limiting characteristics. A possible explanation for this causative linkage is the theory of molecular mimicry, which suggests that similarities between pathogen antigens and platelets act as a mechanism for the transient production of antiplatelet antibodies from the patient's lymphocytes [19]. Exceptions are other infectious agents, such as cytomegalovirus, which may have more chronic and persistent influence on the immune system [20].

The duration of the bleeding symptoms before diagnosis has been reported to determine the course of the disease. Large, retrospective studies emphasized that the most important prognostic factor of chronic ITP is the slow onset of bleeding symptoms [18, 21]. In our study, the results were similar and highlight the importance of the duration of symptoms before diagnosis as an important prognostic indicator for disease progression.

Due to the immunological background of ITP, testing and monitoring for secondary autoimmune diseases are mandatory, especially in chronic and refractory cases. Lowe and Buchanan, in a sample of 126 children aged 10–18 years with ITP, found that 27% of the patients had positive ANA at diagnosis. Of these, 31% had chronic disease and 20% had the acute form [22]. Similar findings were observed in an earlier study in 87 patients with ITP. In this study, 25 children had positive ANA and most of them had chronic ITP [23]. These two studies showed that a significant proportion of patients with positive ANA had also other impaired immunological markers (e.g., anti-double stranded DNA, antiphospholipid antibodies, and anticardiolipin antibodies) and autoimmune-related symptoms. In our study, we found that children with newly diagnosed/persistent form had less frequently pathological immunological markers in contrast to the chronic form, a finding consistent with the previous studies. It must be emphasized that the presence of pathological immunological markers poses a high index of suspicion for secondary causes of ITP, including systemic lupus erythematosus, antiphospholipid syndrome, and Evans syndrome, and these children should be monitored on the basis of this risk. In our study population, a child with chronic ITP was finally diagnosed as Evans syndrome two years after diagnosis.

Platelet count at diagnosis is an important laboratory predictor of the course of ITP. In the study of Lowe and Buchanan, the mean platelet count at diagnosis was 21 × 10^9^/L in chronic cases and 5.5 × 10^9^/L in newly diagnosed ITP [22]. Additionally, Glanz et al. reported that a high number of platelets at diagnosis (>20 × 10^9^/L) combined with the age above 10 years increased the risk of chronic disease fourfold, compared to younger patients with low platelets. Moreover, at the platelet threshold above 30 × 10^9^/L, the same risk was eleven times greater [10]. In agreement with these data, our study showed that most of patients with chronic disease had platelets above 10 × 10^9^/L at diagnosis.

Mucosal bleeding is an important complication of ITP and when it is serious, treatment is required to increase the platelet number. However, several studies as well as ours suggest that, despite its increased incidence and severity, mucosal bleeding is associated with a short disease duration. Elalfy et al. showed that a history of mucosal bleeding at diagnosis is most often associated with acute disease. Specifically, from 224 children with acute disease, 31% had bleeding from mucous membranes, while in the group of 120 children with chronic disease the same proportion was only 6.5% [18]. Similar results were published in earlier studies [10].

The administration of IVIG or corticosteroids, when needed, is a well-documented treatment option in ITP. Data from the Intercontinental Childhood ITP Study Group showed that children with the acute type of the disease had received more often IVIG than the chronic ITP children (32.3% versus 23.8%) [24]. A recent meta-analysis provided similar findings regarding the use of IVIG and corticosteroids and concluded that they may prevent the chronic progression of the disease [15]. Likewise, in our study the percentage of children with newly diagnosed/persistent disease that received IVIG was slightly higher than in chronic disease. Although the immunomodulatory action of IVIG is not fully elucidated, it has been shown to affect both humoral and cellular immune pathways. In ITP patients, IVIG blocks the activation of Fc-receptors in macrophages of the reticuloendothelial system [25, 26]. Since ITP is considered a heterogeneous disease, IVIG may have various effects in different patients, thus explaining why not all patients respond the same way to the treatment.

In conclusion, this retrospective study determined the prognostic characteristics of ITP in children from a large area of Greece. It is confirmed that primary ITP in children is a nonthreatening and self-limited disease, usually lasting less than one year, and chronic form has different prognostic parameters. The use of these parameters can early distinguish children who are expected to have short and uneventful disease duration, in order to minimize their exposure to pharmaceutical interventions. In different case, physicians should be prepared for different diagnostic evaluation and treatment decision options for chronic disease.

## Figures and Tables

**Table 1:** Characteristics between different types of pediatric immune thrombocytopenia.

Parameters	Newly diagnosed/persistent ITP (number 43)	Chronic ITP (number 14)	*p*
Boys	23	7	0.72
Age, years (median, range)	4.8 (1–12)	11.3 (8–16)	0.015
Recent infection	34	3	0.013
Platelet count at diagnosis, ×10^3^/*μ*L (median, range)	14.6 (0–52)	26.3 (0–92)	0.01
Mucosal bleeding	30	7	0.81
Abrupt onset	39	3	0.001
Abnormal immunological markers	5	9	0.003
Treatment with IVIG and/or corticosteroids	40	12	0.78
